# An Empirical Comparison of the Profiles of Security Threat Group Offenders with General Offenders

**DOI:** 10.1177/0306624X221124830

**Published:** 2022-09-29

**Authors:** Christian Leuprecht, David B. Skillicorn, David Bright

**Affiliations:** 1 Royal Military College of Canada, Kingston, ON, Canada; 2 Queen’s University, Kingston, ON, Canada; 3 Deakin University, Melbourne, VIC, Australia

**Keywords:** offenders, gangs, violence, substance abuse, risk stratification

## Abstract

Datasets of offender attributes, both pre-custody and in-custody, were provided by the Correctional Service of Canada with the goal of exploring whether Security Threat Group (STG) offenders (informally, gang members of various kinds) differ in any systematic way from other offenders. For pre-custody attributes, we show that the entire offender population varies along two almost independent axes, one associated with affinity for violence, and the other with affinity for substance abuse. Within this structure, STG offenders are characteristically less extreme, in either direction, than the general offender population. For approximately two dozen attributes, STG offenders, as a group, tend to have higher values; for a few, they tend to have lower values. For in-custody attributes, the entire offender population forms a triangular structure whose vertices represent: passivity; violence and troublemaking; and involvement in programs leading to partial release. The differences between the STG offender population and the general offender population are small. An offender who is placed at the high end of the propensity for violence axis and/or the high end of the substance abuse axis based on pre-custody attributes is much more likely to be involved in incidents, grievances, and violence while in custody. This may have implications for risk stratification of incoming offenders.

Research has tended to divide the study of organized crime groups between those operating in society at large, and those operating within correctional settings (“prison gangs”) (see Pyrooz et al., 2011). The Canadian Criminal Code section 467.1(1) defines an organized crime as a serious crime committed by three or more people, so that even quite small-scale criminal activities qualify as organized crime. The definition of a Security Threat Group (STG) in a Canadian correctional context is “any formal or informal ongoing inmate/offender group, gang, organization or association consisting of three or more members” (Commissioner’s Directive, 2016). Affiliation with an STG is considered a risk factor for offender management. STGs are categorized as: street gangs, prison gangs, outlaw motorcycle gangs, traditional organized crime, indigenous gangs, white supremacy groups, subversive groups, terrorist organizations, and hate groups. These categories reflect the extent to which STGs in correctional settings largely reflect affiliations formed in the outside world. In the United States, estimates of gang affiliation within prisons routinely sit above 10% (e.g., Hill, 2009; National Gang Intelligence Center, 2011; Pyrooz & Mitchell, 2020; Wells et al., 2002; Winterdyk & Ruddell, 2010). An estimate in 2016 places the figure at 15% (Pyrooz & Mitchell, 2020) whilst the highest estimate is 19% (Winterdyk & Ruddell, 2010). In a recent review of the prevalence literature Decker et al. (2022) claim that it is “reasonable to conclude that around one out of every 7 of the 1.5 million people incarcerated in federal and state prisons are associated with a gang” (p. 312).

Data on gang membership are routinely collected and utilized by prison authorities (e.g. for the purposes of risk assessment, decisions regarding restrictive housing, access to custodial programs; Pyrooz et al., 2020). Notwithstanding the popularity of such data among prison authorities, gang researchers are traditionally skeptical of the validity of this data. In an assessment of the validity and utility of gang membership administrative data, Pyrooz et al. (2020) compared data from an extensive interview-based survey on gang membership with administrative data and found a strong concordance between the two. Nonetheless, of relevance to the current study, administrative data tends not to do as well in accurately capturing misconduct in prisons (e.g., Pyrooz et al., 2020; Steiner & Wooldredge, 2014).

The aggressive and violent nature of many STGs is thought to pose a safety concern for both prisoners and correctional officers. For example, Camp and Camp (1985) evaluated 22 U.S. state prisons and found that STG members represented only 3% of the prison population, but were involved in 50% of violent incidents in prison. This violent behavior not only poses a risk to inmates and prison officials but can also be disruptive to rehabilitative efforts such as housing and work assignments (Griffin & Hepburn, 2006). Fischer (2001) conducted a study of STG members in prison and discovered that STG members were 74% more likely to violate discipline codes compared to their non-STG counterparts. More recent research confirms that imprisoned gang members have higher rates of misconduct and victimization compared with former gang members and those who have never been gang members (Cihan & Sorensen, 2019; Decker et al., 2022). Concomitantly, gang members are also overrepresented in restrictive housing in US prisons (Pyrooz & Mitchell, 2020).

Much like the broader research literature on orgnaized crime, the literature on organized criminal groups (OCGs) suffers from definitional issues (see Finckenauer, 2005; Kirby & Snow, 2016. Many papers fail to delineate the particular kind of groups they are studying, failing to distinguish between: organized crime groups with a strong hierarchical structure (e.g. mafia, terrorist groups), groups with a functional criminal purpose but a gang ethos (e.g. street gangs smuggling guns), and groups that are anti-social, with criminality as one facet of that attitude (e.g. outlaw motorcycle gangs, youth gangs, hate groups). Within a correctional context, research has often focused on “prison gangs,” without making distinctions about the kind of groups that such gangs reflect (see Lessing, 2016).

Definitional issues also make it difficult to estimate membership of organized crime groups, quite apart from the uncertainties arising because of the incentives for non-members to fake membership (to gain respect) or for members to conceal their membership (to avoid law-enforcement interest). In Canada, in 1996, approximately 2% of the offender population was considered to belong to an STG; by 2003 it was 5% (Nafekh & Stys, 2004); by 2010, 10% (Mackenzie, 2012) and at present, 21%. In the U.S., a study in 2006 found that STG members were nearly 13% of the prison population (Ruddell et al., 2006). This growth continued in 2010, with 19.1% of the prison population identifying as STG-affiliated (Winterdyk & Ruddell, 2010). Allowing for uncertainties in exactly what is being counted, Canada and the U.S. exhibit broadly consistent levels of STG involvement and similar growth rates. This growth probably represents a combination of real growth in the number and size of organized crime and STG activities, changes in the law defining organized crime offences,^1^ and better elicitation and labeling of those offenders meeting STG criteria.

There have been many attempts to characterize STG members in terms of their demographics and personal attributes. Much academic work (Lafontaine et al., 2005; Mitchell et al., 2017) takes a population of individuals convicted of organized crime offenses and looks at possible contributing factors: individual risk factors such as low self-esteem, family risk factors such as poverty and child abuse, community risk factors such as high crime rates, and peer group risk factors such as having friends who are STG members. They fail to control for similar individuals who do not meet STG criteria, and who surely share many of the same characteristics.

Most large-n studies of STGs have been conducted in the US, where state prisons have had to deal with extensive STG networks. These large-n research studies investigate the purpose of STGs and incentives for STG members to join. In these investigations, the Texas Syndicate and La Nuestra Familia are often cited as historical and in-depth case studies (Buentello, 1986). Some of the large-n case studies compare street gangs to prison STGs to determine if one has any influence over the formation of the other (Wood et al., 2014). These two groups are often compared to determine if members share common characteristics (Pyrooz et al., 2011).

Much of the academic discussion surrounding STG activity in Canada has been limited to disputes over the proper definition of a STG, the precursors for STG involvement, and what constitutes STG membership (Ezeonu, 2014). There is a signifucant focus within the Canadian literature on the structure of Canadian street gangs and the danger these groups can pose to cities and public safety (Ruddell et al., 2009).

Research posits several attributes according to which STG members differ from other offenders in a correctional context. These attributes are summarized in this list:

- Age. STG members in the U.S. are 5 years younger than other offenders (Rufino et al., 2012) but only a year younger in Canada (Scott & Ruddell, 2011).
- Previous criminal history. STG members are more likely to have been arrested and initiated into criminal behavior at a young age than non-STG members (Griffin & Hepburn, 2006).
- Attitudes. A U.K. study shows that the attitudes of STG members differed in areas of social status, social dominance, and dislike for authoritative influences (Wood et al., 2014); A U.S. study showed that STG member more actively pursue positions of social status (Camp & Camp, 1985).
- Personality differences. A U.K. study found that STG members often had low agreeability scores, were not conscientious, and were highly impulsive (Egan & Beadman, 2011).
- Education. In the U.K., STG members are less likely to have graduated high school or achieved an equivalent level of education (Egan & Beadman, 2011). In the U.S., 19% of non-STG members had a High School Diploma, but only 16% of prison STG members (Rufino et al., 2012).
- Marital status. U.S. STG members are less likely to be married or have children than other offenders (Krienert & Fleisher, 2001). 64% of STG members had never married, compared to 49% of other offenders (Rufino et al., 2012). In Canada, Scott and Ruddell (2011) found that 49% of STG members are single compared to 59% of other offenders. These differences may arise from differences in the requirements for common-law marriages and in the differing incentives of social assistance systems.
- Employment. In the U.S. 57% of STG members were employed prior to incarceration, compared to 83% of other offenders (Rufino et al., 2012). Only 10% of STG members earned $25,000 annually while 25% of other offenders earned at least this much prior to incarceration (Krienert & Fleisher, 2001).
- Arrest record. Fleisher and Decker (2001) found that 32% of STG members had 15 or more arrests compared with 7% for non-members. 75% of STG offenders were arrested as a juvenile, compared to 41% of other offenders (Rufino et al., 2012). STG members also had more prior youth convictions than their non-STG counterparts (Scott & Ruddell, 2011). 73% of STG members had one or more prior adult convictions, compared to 65% of other offenders. STG members were more likely to serve longer sentences. 0.6% served 10 or more years (0% non-STG), 2% served 5 to 10 years (0.8% non-STG), and 65% of prison STG members served 1 day to 5 years (49% for non-STG).
- Drug use. In one study, nearly 90% of STG members interviewed admitted to using marijuana, while only 66% of non-STG members used it (Krienert & Fleisher, 2001). In contrast, non-STG offenders were more likely to use crack cocaine (45%); many STG members avoided consuming the drug because of the stigma attached (Wilditz et al., 2007 ) but a U.S. national survey found that 66% of STG members sold crack cocaine (Decker et al., 2008).
- Alcohol abuse. 57% of STG members reported alcohol abuse compared to 38% of other offenders (Wilditz et al., 2007).
- Violence. STG members are more violent than non-STG members. Krienert and Fleisher (2001) found that STG members were more likely to have been involved in assault and robbery. This pattern of aggression continued during incarceration. Fleisher and Decker (2001) found that STG members were 74% more likely than non-STG members to commit disciplinary violations. Additionally, STG members assaulted other inmates more often than non-STG members. In another study of assaults in Texas prisons, STG members were three times more likely to commit assault (DeLisi et al., 2013).
- Gang tattoos. Only 38% of male STG members have gang tattoos when they enter, and only 7% of female STG members (Knox, 2005).
- Religious activity. In the U.S. 51% of offenders attend religious services (Knox, 2005) but 69% of prison officials believed that “inmates attempted to use religious services as a front for a Security Threat Group or STG.”

Many items on the list are consistent with known risk factors for general and violent offending. For example, the top eight risk factors (referred to as the “central eight”; see Bonta & Andrews, 2007) includes the “big four” (history of antisocial behavior, antisocial personality, antisocial congitions, antisocial associations) and the “moderate four” (substance abuse, poor family marital and relationships, poor school and employemnt record, few or no prosocial recreational activities).

This article analyzes the characteristics of the offender population, using attributes available to and collected by CSC, using an inductive methodology, without generating hypotheses about differences between STG and non-STG offender populations. The results can be compared to the patterns obtained by other methods, and in other countries.

## Data

Two datasets were generated by CSC personnel, describing 11,414 incarcerated offenders (excluding those under community supervision, typically about 40% of the offender population at any given time). The first dataset contains attributes collected at the time of incarceration, during the Offender Intake Assessment. These include: previous criminal history (including previous sentences), demographic information, and assessments made by CSC personnel from intake interviews. This dataset contains values for 367 attributes. The second dataset contains attributes collected during incarceration. These include: involvement in programming, institutional incidents, offender grievances, offender movement (security placements and transfers), and visits and correspondence. This dataset contains values for 284 attributes. The meaning and collection protocol for both sets of attributes collected by CSC personnel can be found in Appendix A of Brown and Motiuk (2005). These are supplemented by demographic and personal history attributes. A third set of attributes for STG offenders alone was extracted, containing information about the specific group in which each offender was involved (where known), and the role played in the group. When an offender is, or had been, a member of more than one STG, data was provided about the first and last group (covering the vast majority of offenders since membership in more than two groups is rare). Data for this set of offenders was previously used in (Skillicorn et al., 2015) but the data was updated in 2016 with a larger set of attributes and STG membership data.

2,475 of the 11,414 offenders are labeled as having an organized crime affiliation if: they were convicted of an organized crime offense, or were identified as having a STG affiliation. This represents 21.7% of the entire offender population. Details of the demographics of this offender population, and the STG groups to which they belong can be seen in Tables 1 and 2. It is clear that STG membership skews younger but otherwise the differences are small.

**Table 1.** Demographic Comparison of the Offender Population.

	Non-STG offenders	STG offenders
Count	8,939	2,475
Male	8,575 (96.0%)	2,408 (97.3%)
Female	364 (4.0%)	67 (2.7%)
% Any violent offense	71.5%	70.4%
% Any non-violent offense	26.5%	27.4%
Avg number of federal sentences	1.67	1.58
Age distribution		
70+	1.5%	0.2%
60+	4.6%	1.3%
50+	14.2%	4.9%
40+	26.0%	14.0%
30+	29.0%	33.4%
20+	24.0%	45.4%
Under 21	0.6%	0.7%

**Table 2.** Types of STGs in the Offender Population and Their Levels of Membership.

STG type	Members on intake	Members joined in custody
Street gangs	532	234
Aboriginal gangs	308	101
Motorcycle gangs	166	39
Traditional organized crime	50	18
Prison gangs	36	28
White supremacist groups	22	10
Terrorist organizations	8	1
Asian gangs	8	18
Racial extremist organizations	2	1
East European organized crime	1	0

## Methods

The methods of analysis were used in a previous project examining radicalization in CSC (Skillicorn et al., 2015), with some newly developed technical improvements. For each of the datasets, the following process was followed:

1. Preprocessing and hashing techniques developed by the authors were used to map attribute values to numeric values in ways that preserved, as far as possible, the semantics underlying them. This enables numeric techniques to be applied to cluster the data. A newly developed smoothing technique was applied to reduce the disortions introduced when raw attribute values had a wide range of potential values that were sparsely populated.
2. Some selection of attributes was carried out. A group of attributes measuring intellectual and social disabilities, were removed. Their presence creates a strong clustering between a small population that might be characterized as special needs, and the wider offender population. The remaining pre-custody attributes were divided into two subsets: those that describe demographic and personal attributes of offenders; and those that describe criminal history.
3. Datasets were clustered using singular value decomposition (SVD), an algorithmic clustering technique that discovers variation. The coding process produces matrices whose rows correspond to offenders and whose columns correspond to attributes. These matrices can be used to visualize both offenders and attributes, and to calculate similarities among them. SVD is related to Principal Component Analysis, and has the effect of rotating the cloud of points corresponding to either records or attributes in such a way that the variation among them is maximized in three-dimensional space. In the figures that follow, points corresponding to each offender are plotted in two dimensions. Points that are close represent offenders with similar characteristics and variation across the figures represents consistent variation involving multiple attributes.
4. SVD considers variation among records (offenders) and attributes simultaneously. If points corresponding to offenders and points corresponding to attributes are plotted in the same space, then it is meaningful to consider each offender point as being pulled toward attribute points for attributes for which it has large values, and attribute points as being pulled toward offender points that have large values for it. The SVD plot represents the stable positions achieved by this process. This enables variation in offenders to be explained in terms of variation in attributes.
5. The effects of attributes on the variation among offenders can be leveraged to understand, in greater detail, which attributes make the greatest contribution. For example, a particular attribute can be weighted by a factor of 2, and the resulting change in offender variation calculated. An attribute that strongly influences offender variation will cause a large change.

Several prediction techniques were also used to examine whether pre-custody attributes are predictive of in-custody properties. In all cases, the available data is randomly divided into a training and a testing set, a predictive model is built from the training set and evaluated on the testing set. Support vector machines contruct the optimal hyperplane that separates two classes using an optimization technique; random forests use an ensemble of decision trees, each of which is learned from a subset of the training data, and predictions are made based on the votes of each tree given an example from the training set; and neural networks use a two-layer network of devices that were inspired by neurons to compute a non-linear function of the input attributes provided. These three techniques are known to perform well on many kinds of data.

## Results

### Pre-custody Analysis

**Figure 1.**
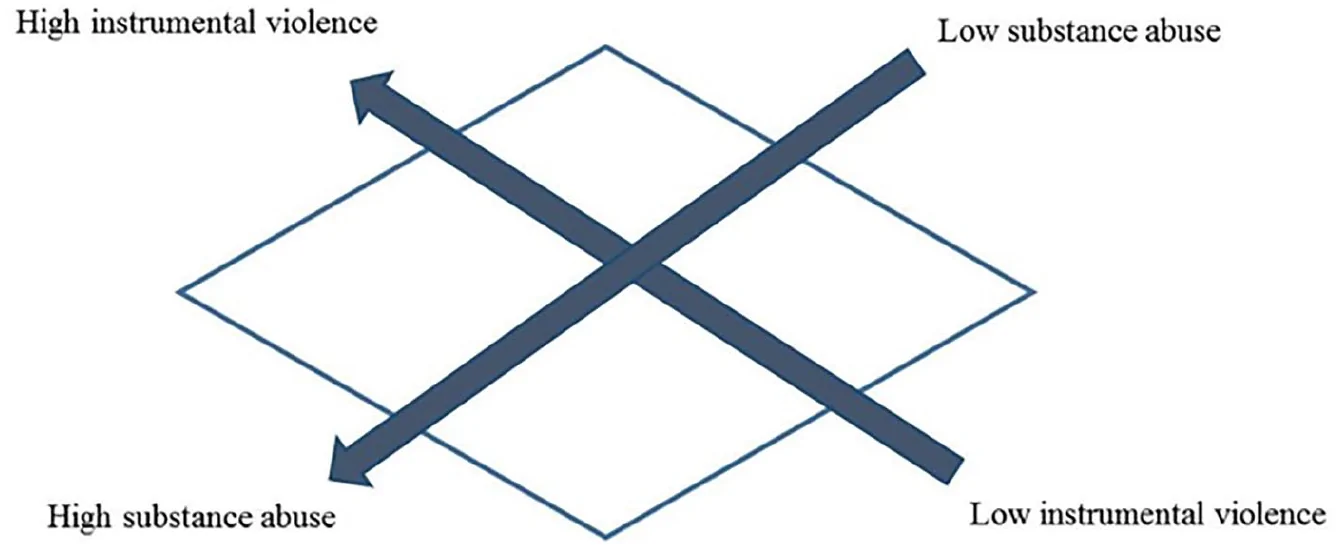
Schematic representation of the variation across the offender population based on selected pre-custody attributes.

We begin with the personal and demographic attributes. The overall variation in the total offender population based on these attributes is shown schematically in Figure 1. The shape shows that STG offenders (clusters) are no different from other offenders; rather offenders form a single cluster which has a diamond-like shape. Within this shape we can infer which attributes are responsible for the variation. In this case, variation occurs in two almost-orthogonal directions, as shown by the arrows (hence the diamond shape). One direction captures variation with respect to attributes associated with violence; therefore, points on the top left correspond to offenders with an affinity for violence and points on the lower right correspond to offenders with an antipathy to violence. The other direction captures variation with respect to attributes associated with substance abuse. Points on the lower left correspond to offenders with an affinity for substance abuse and, on the upper right, offenders with an antipathy to substance abuse. These variations are almost independent so that there are, for example, offenders who are high on affinity for violence but low on substance abuse, high on affinity for violence and also high on substance abuse, and so on. However, individuals who are at the left-hand point of the diamond (high affinity for both violence and substance abuse) are especially problematic from a corrections perspective. Individuals at the right-hand point of the diamond (low affinity for violence and substance abuse) are the least likely to cause problems in custody.

Both STG offenders and the general offender population show the same clustering, There are two main differences between the two populations. First, STG offenders are less extreme compared with the general population. The structure of STG offenders forms a crisp diamond shape, while the general population structure is slightly more elliptical. In other words, those who are extreme in both dimensions (high affinity for violence, low affinity for violence, high affinity for substance abuse, low affinity for substance abuse) tend to be non-STG offenders.

Second, there are differences in the distributions of values for some attributes. When this happens, the STG offender population is typically more skewed than the general offender population. This is not an effect of the smaller size of the STG offender group, since this would be detected as a sparser diamond structure for that population.

### Visualizing the Two Groups of Offenders

The differences between STG and other offenders can be visualized by plotting points that correspond to each offender and color-coding them by the corresponding values of a particular attribute. The following figures show the diamond structure, for STG offenders (on the left) and for other offenders (on the right). To fit both into a single figure, the diamond shapes are visually compressed horizontally.

**Figure 2.**
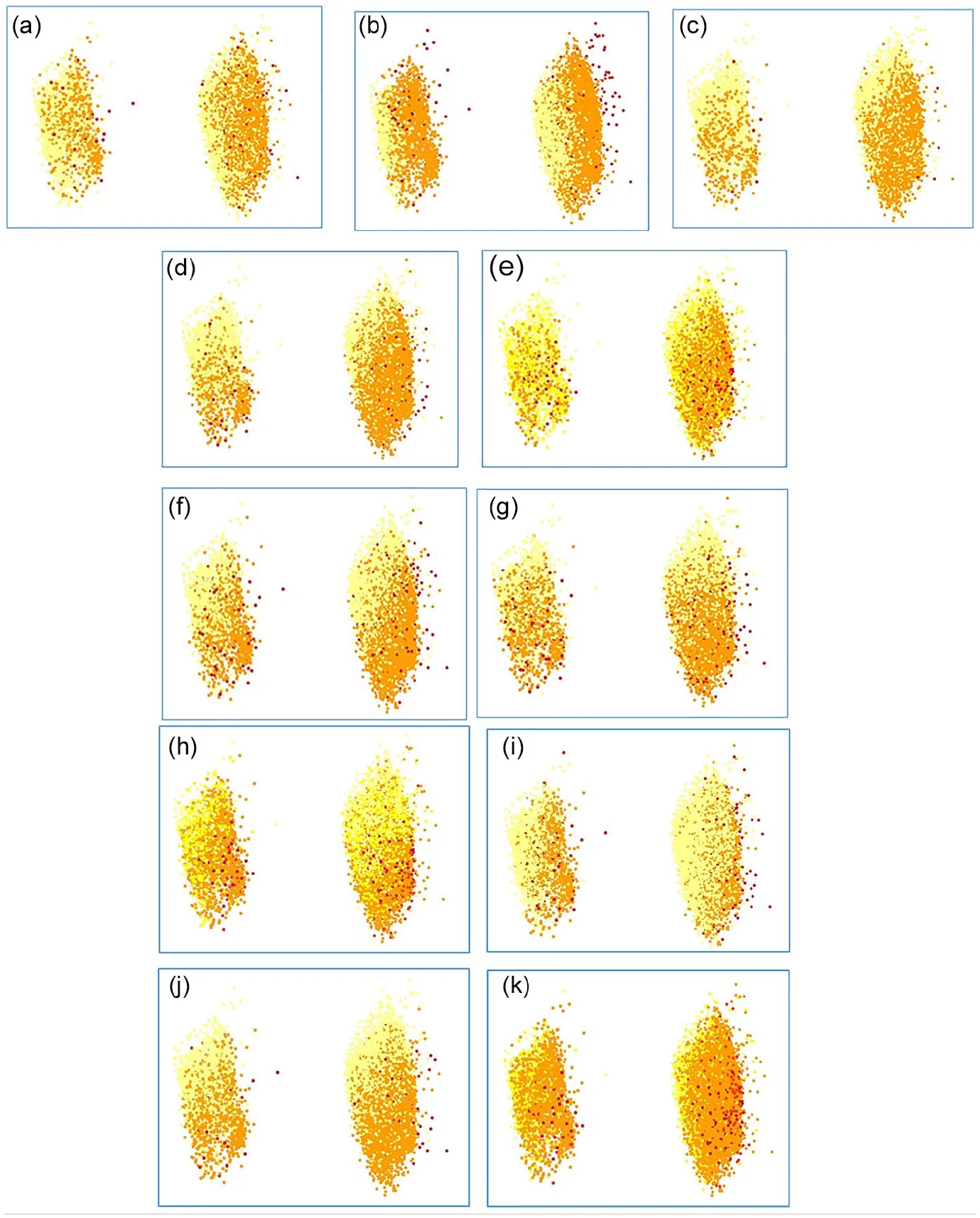
(a) Variation for the STG (left) and others (right), color coded by attribute magnitude, unemployed at time of arrest, (b) variation for the STG (left) and others (right), color coded by attribute magnitude, alcohol use interferes with employment, (c) variation for the STG (left) and others (right), color coded by attribute magnitude, negative toward the criminal justice system, (d) variation for the STG (left) and others (right), color coded by attribute magnitude, supportive of instrumental violence, (e) variation for the STG (left) and others (right), color coded by attribute magnitude, attitudes domain score, (f) variation for the STG (left) and others (right), color coded by attribute magnitude, has low frustration tolerance, (g) variation for the STG (left) and others (right), color coded by attribute magnitude, manipulates others to achieve goals, (h) variation for the STG (left) and others (right), color coded by attribute magnitude, displays narrow and rigid thinking, (i) variation for the STG (left) and others (right), color coded by attribute magnitude, community function domain score, (j) variation for the STG (left) and others (right), color coded by attribute magnitude, family function domain score, and (k) variation for the STG (left) and others (right), color coded by attribute magnitude, has difficulty coping with stress.

Figure 2a (unemployed at time of arrest) and Figure 2b (alcohol use interferes with employment) show some of the attributes associated with the affinity for substance abuse axis. Lighter colors correspond to higher values of the property concerned. These visualizations illustrate the relationship between the distribution of each specific attribute and the global variation that produces the diamond shape. The lighter colored points are strongly represented along the lower left side of the diamond structure; therefore, we infer that offenders who have higher values for, say, unemployment are also offenders who have higher values for the constellation of attributes associated with substance abuse. There are also differences in the distribution of colors between the left and right diamonds. Both populations (STG and other offenders) show the same association between (un)employment and substance abuse, but the distribution of, say, light-colored points is different, indicating that the distribution of values differs between the two sub-populations. We define this dimension as “lifestyle instability,” characterized by low levels of education, poor employent history, and drug abuse. Lifestyle instability factors such as poor work histories, drug and alcohol abuse have been associated with general and violent offending.

Figure 2c negative toward the criminal justice system, Figure 2d supportive of instrumental violence, Figure 2e attitudes domain score, Figure 2f has low frustration tolerance, Figure 2g manipulates others to achieve goals, and Figure 2h (displays narrow and rigid thinking) show some of the discriminating attributes associated with the affinity for violence axis. Lighter colors are strongly represented along the upper left side of the diamond structure. We define this dimension as “antisocial attitudes and behavior” including antisocial attitudes and thinking patterns generally supportive of criminal behavior.

Figure 2i community function domain score, Figure 2j family function domain score, and Figure 2k has difficulty coping with stress are some examples that are associated with the left-hand point of the diamond structure, that is with high levels of affinity for *both* violence and substance abuse. We interpret this dimension as a combination of poor interpersonal skills and poor coping skills. These deficits have been shown to be associated with increased risk of general and violent offending.

These figures show the attributes that are associated with, and so drive, the observed variation across the entire offender population. It is not surprising that attitudes to instrumental violence are associated with an affinity for violence. In addition, some research demostrates a link between narrow and rigid thinking and risk of general and violent offending (see Blanchette, 1997; Ross et al., 1988).

We now focus on those attributes for which the distribution of values differs between these two sub-populations. These are the attributes that most strongly differentiate STG offenders from other offenders (although we already know, from the global structure, that these differences cannot be large). Table 1 summarizes the attributes for which the STG and general offender populations have different distributions. The first block describes those attributes for which STG offenders tend to have higher values than the general offender population, dividing them into those associated primarily with lifestyle instability (tending to cause such offenders to be placed toward the lower left), those associated primarily with antisocial attitudes and behavior (tending to cause such offenders to be placed toward the upper left), and those associated with poor interpersonal skills and poor coping skills (tending to cause such offenders to be placed close to the left-hand point of the diamond). The second block describes attributes for which STG offenders tend to have lower values than the general offender population.

The domain scores are constructs used by CSC and represent aggregates of all of the attributes of a particular kind. The prevalence of these attributes in the table shows, first, that these aggregate scores are good surrogates for the finer-grained attributes they represent and, second, that most individual attributes within a domain are aligned in the same direction, even if weakly, so that their aggregate attributes are revealing when the individual attributes that contribute to them are, singly, too weak to do so (Table 3).

**Table 3.** Attributes Associated With High Affinities in the Diamond Structure and Different Distributions for the Two Offender Groups.

General lifestyle instability	Antisocial attitudes and behavior	Poor interpersonal and coping skills
*Stronger for STG offenders*		
Associates with substance abusers	Negative attitudes to criminal justice system	Associates domain score
Disrespectful of property (weak)	Supportive of instrumental violence	Marital/family domain score
Employment domain score	Attitudes domain score	Childhood lacked family ties (weak)
Unemployed at time of arrest	Any violent offense	Has no parenting responsibilities
Substance abuse domain score	Aggression issues	Poor parental relations as a child
Has tattoo (weak)	Empathy skills are limited	Witnessed family violence during childhood
Financial difficulties	Has low frustration tolerance	Impulsive
	Has difficulty solving interpersonal problems	Personal/emotional domain score
	Manipulates others to achieve goals	Has difficulty coping with stress
	Mental health intervention, past or present	Birthdate
	Displays narrow and rigid thinking	Race
	Displays non-conforming attitudes toward society (weak)	Number of federal sentences
	Risk taking, thrill seeking (weak)	Sentence number
	Time management skills are problematic	
*Weaker for STG offenders*		
Citizen-country-code	Sexual offense	Unstable or absent work history
All attributes involving substance abuse with alcohol	Current sex offense resulted in death or serious harm	Overall-dynamic-factor-e
		Overall-static-factor-e
		Age at sentencing

The attributes labeled as weak have visible variation for STG members but much less so than the other attributes in the table.

Attributes that do *not* have a different distribution between the two groups, but might have been expected to, are:

- no high school diploma
- family members criminally active during childhood
- assertiveness skills are limited
- ability to generate choices is limited
- ability to link actions to consequences is limited
- difficulty setting goals

Those attributes associated with the affinity for violence axis and for which STG offenders have higher values are attributes of attitudes and personality, rather than social environment. The research literature has often suggested that environmental factors are associated with organized crime and STG membership; if there is such an environmental effect, it seems to be indirect.

The attributes associated with lifestyle instability include substance abuse (e.g., associates with substance abusers), consequences of substance abuse (unemployed at time of arrest), or both (financial difficulties, disrespectful of property).

### Attributes Specific to STG Offenders

The attributes specific to organized crime offenders can also be overlaid on the diamond structure, plotted only for these offenders, showing how specific STG activity properties are related to the variation among these offenders. However, relatively few of the STG offenders were actually convicted of organized crime offenses, and precise information about, for example, roles is difficult to obtain. The blue points in the figures correspond to the unknown or not applicable cases.

**Figure 3.**
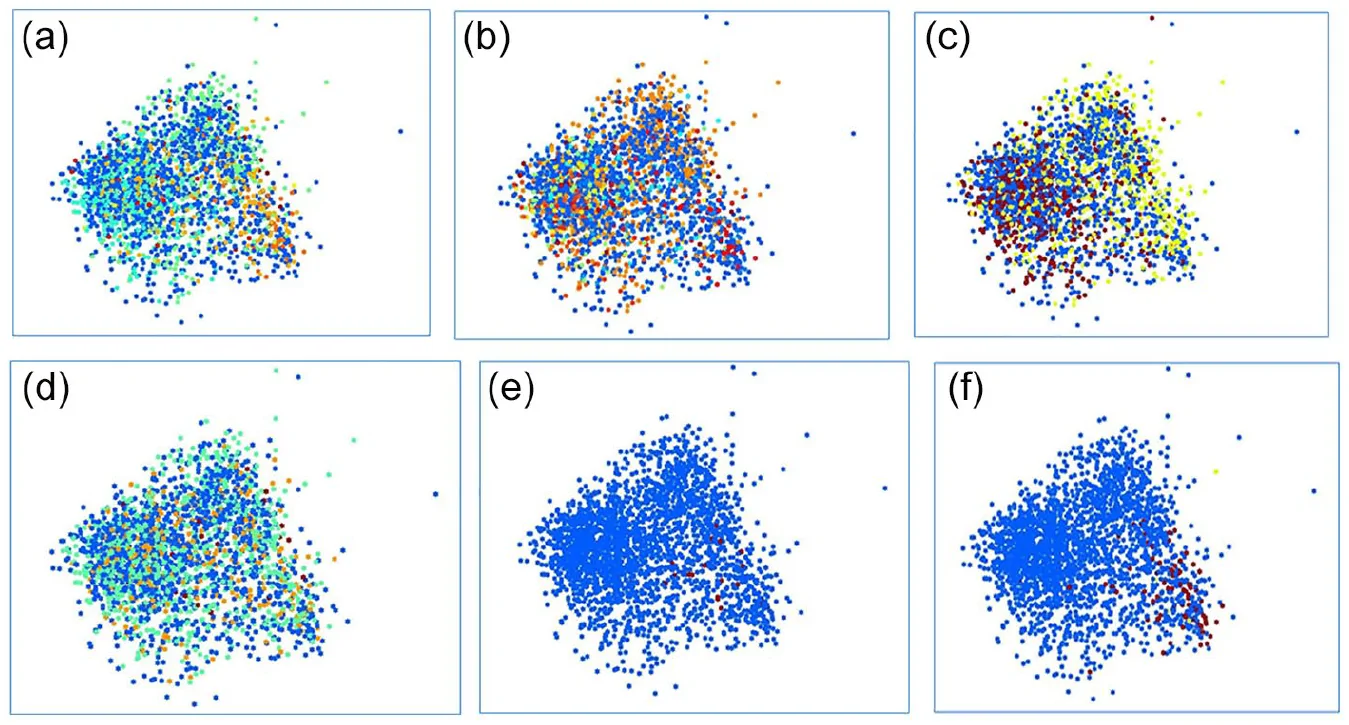
Group affiliations, roles, and activities: (a) group affiliation, when known (yellow-orange points correspond to traditional organized crime offenders), (b) subgroup affiliation, when known, (c) activity level within the group (yellow—active), (d) role within the group, (e) convicted of an offense of instruction (telling someone else to commit a crime), and (f) convicted of an offense of commission.

Figure 3 shows the group affiliations overlaid on the diamond structure (using a different color coding to make it easier to see the relatively rare points against the background) with the same orientation—affinity for violence at the upper left and affinity for substance abuse at the lower left. Although members of most groups can be found anywhere in the diamond structure, there are clearly some groups whose members are low on affinity for violence and affinity for substance abuse. These groups turn out to be the traditional organized crime groups (e.g., mafia).

Figure 2 shows that there is little structure when group membership is refined below the level of the type of group. Subgroups are groups within a particular kind of organized crime group (traditional, motor cycle, youth, indigenous) that have a particular identity. The figure shows that such subgroups are heterogeneous, that is, they do not form from individuals who have similar attribute values on either the propensity for violence or propensity for substance abuse axes. The brown-colored points are mostly absent from the right-hand point of the diamond, but they are present everywhere else; otherwise points of the same color seem evenly distributed across the diamond. This may suggest that “successful” groups require members across a wide range of attribute values to be effective. For example, such groups may need an enforcer (with an affinity for violence) but not everyone in the group needs to have this attitude.

Figure 2c shows that activity level is negatively associated with affinity for substance abuse—the yellow points are almost entirely across the top right edge of the diamond structure. In other words, inactive members are more likely to be substance abusers, although we cannot conclude in which direction causality might apply.

Figure 2d shows that role within the group has no particular association with position within the diamond structure. This is surprising since, in other organizations, personal and demographic attributes tend to correlate with the kind of function an individual carries out for that group.

Figure 2e and f show those convicted of the offense of instruction to commit a crime on behalf of a criminal organization (Criminal Code section 467.13), and those convicted of commission of a crime on behalf of a criminal organization (Criminal Code section 467.12). Both groups of individuals are placed toward the right-hand end of the diamond structure—those with low affinity for violence and low affinity for substance abuse, in the case of commission notably so. There are several potential explanations: those with these characteristics may be more functional than their more-violent or more-substance-abusing peers and so commit more significant crimes; those with these characteristics may be easier to prosecute for organized crime offenses; or it may represent a correlation of these characteristics with traditional organized crime groups who are predominantly in this area of the diamond, and might prefer members with these characteristics.

### In-custody Analysis

We now turn to the in-custody attributes, those that reflect what happens to offenders while they are incarcerated. The pre-custody attributes can reasonably be assumed to be independent variables—they are the results of historical factors and demographics. In-custody attributes, by contrast, are a mixture of independent attributes (choosing to cause an incident or file a grievance), and dependent attributes (visitors, involuntary segregation). It is important, when considering attributes that differ between groups, to distinguish whether STG offenders behave differently, or are treated differently since this has different implications for offender management of the two groups.

**Figure 4.**
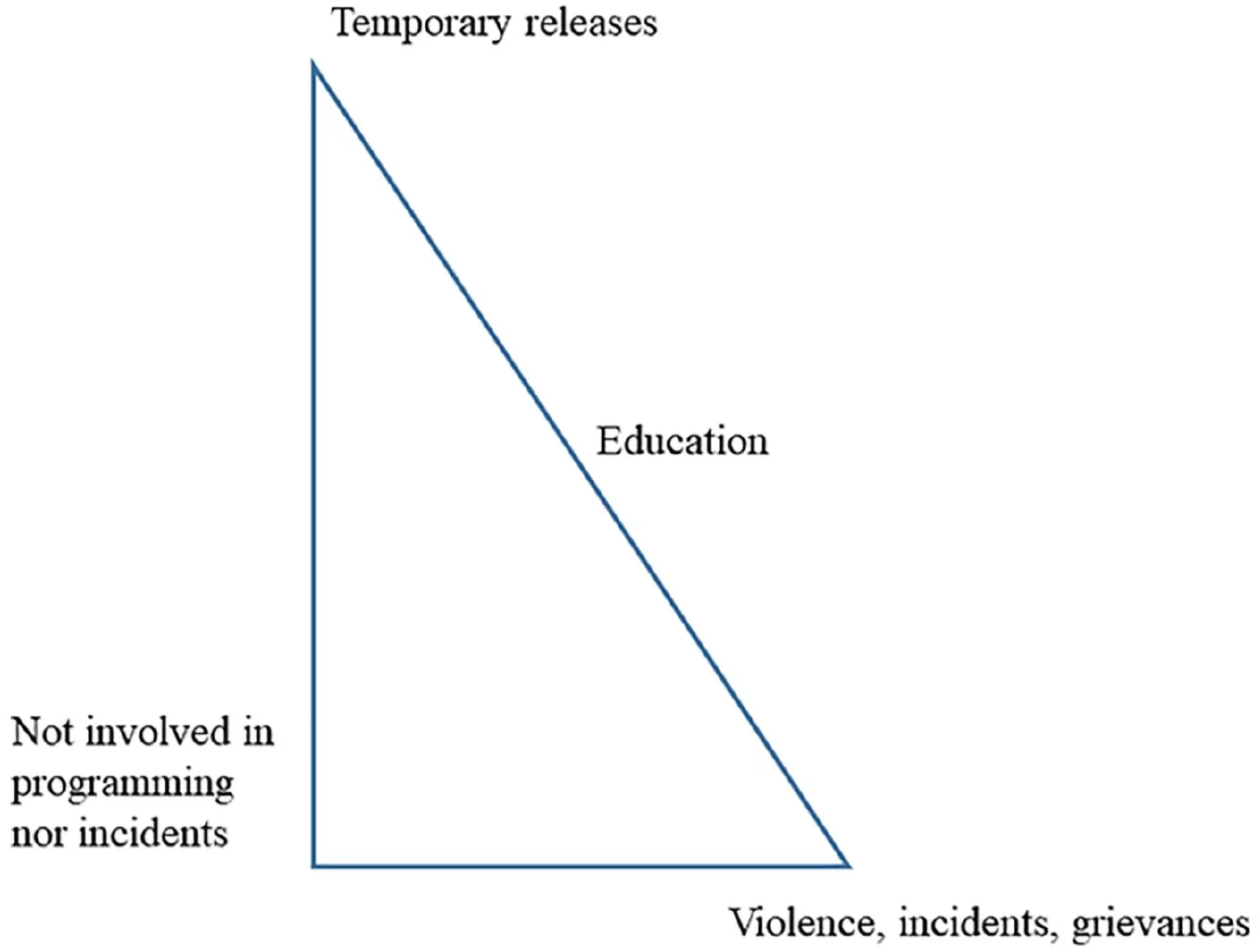
Schematic representation of the variation across the offender population based on in-custody attributes.

The structure of variation for the in-custody variables is triangular, as shown in Figure 4. The default offender who does not participate in programming nor become involved in incidents is placed close to the left-hand lower corner. With respect to this neutral position, variation occurs in three directions: vertically, associated with various programs that can lead to temporary release, up and to the right, associated with educational programming of various kinds, and to the right, associated with negative issues—incidents, grievances, and violence.

**Figure 5.**
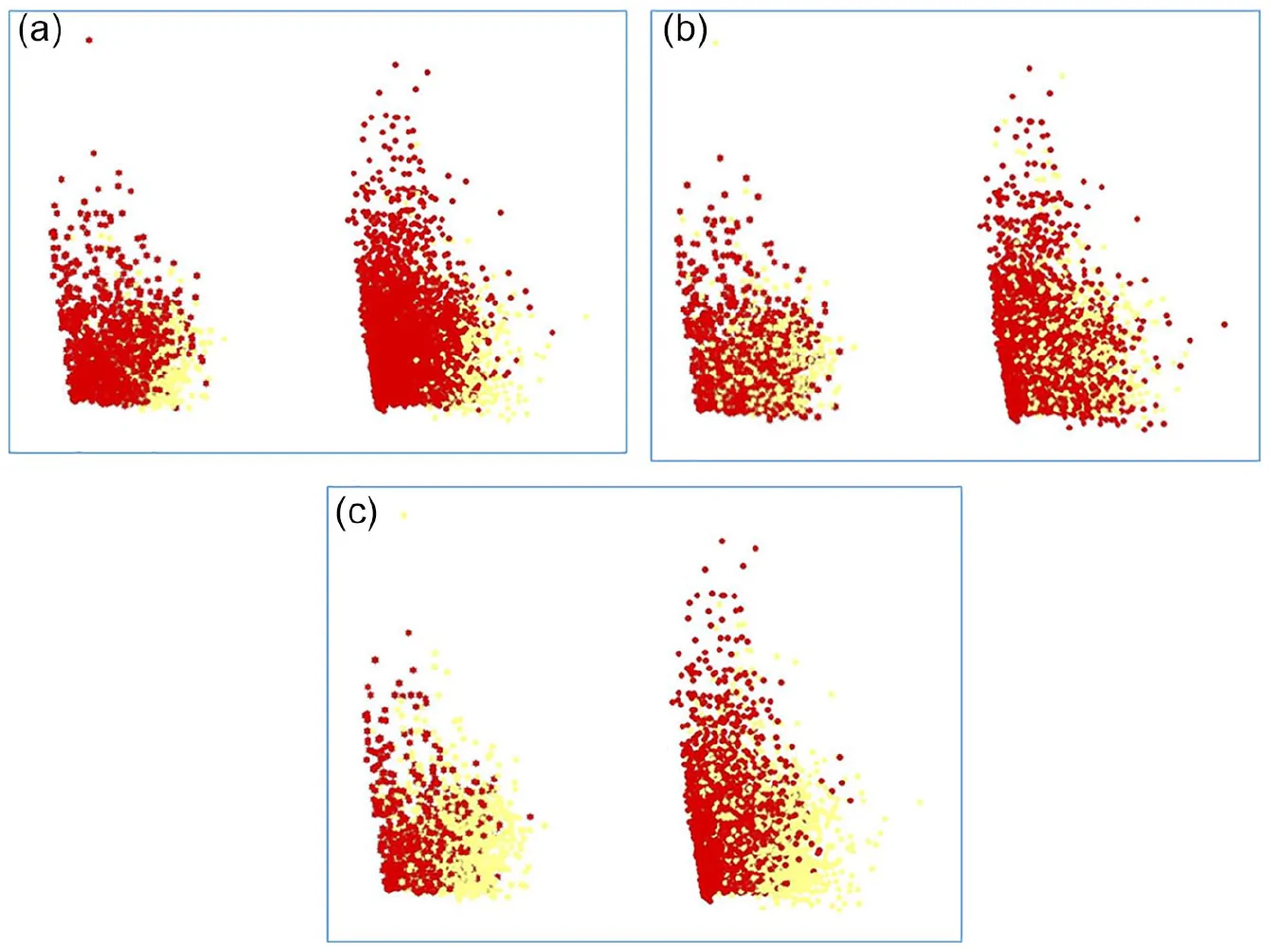
(a) variation for the STG (left) and others (right), color coded by attribute value, charges-serious (yellow = more charges), (b) variation for the STG (left) and others (right), color coded by attribute value, any grievances (yellow = more grievances), and (c) variation for the STG (left) and others (right), color coded by attribute value, any incidents (yellow = more incidents).

Figure 5 show the distribution of values for attributes related to difficulties, challenges and concerns while in custody for the two groups of offenders. As before, there are two kinds of differences: in the shape of the triangle structure and in the distribution of values between the two sub-opulations. The left and right plots for these figures are similar, showing that both kinds of differences between STG offenders and other offenders are small.

Table 4 lists those attributes that have the greatest differences in distribution. There are also small differences in the values of first-placement-location and intake-location that are probably due to the distribution of STG groups across Canada. For example, Nafekh and Stys (2004) noted that outlaw motorcycle gangs and traditional organized crime groups operate primarily in Montréal and Québec City, and so are likely to be sentenced and placed in Québec institutions.

**Table 4.** In-Custody Attributes With Different Distributions for STG Offenders.

*S*tronger for STG offenders
Highest pay level
Number of segregation admissions in first year of incarceration
Number of days spent in segregation in first year of incarceration
Weaker for STG offenders
Any self injury
Education completed
Dangerous offender flag

### Predicting In-Custody Behavior From Pre-Custody Attributes

**Figure 6.**
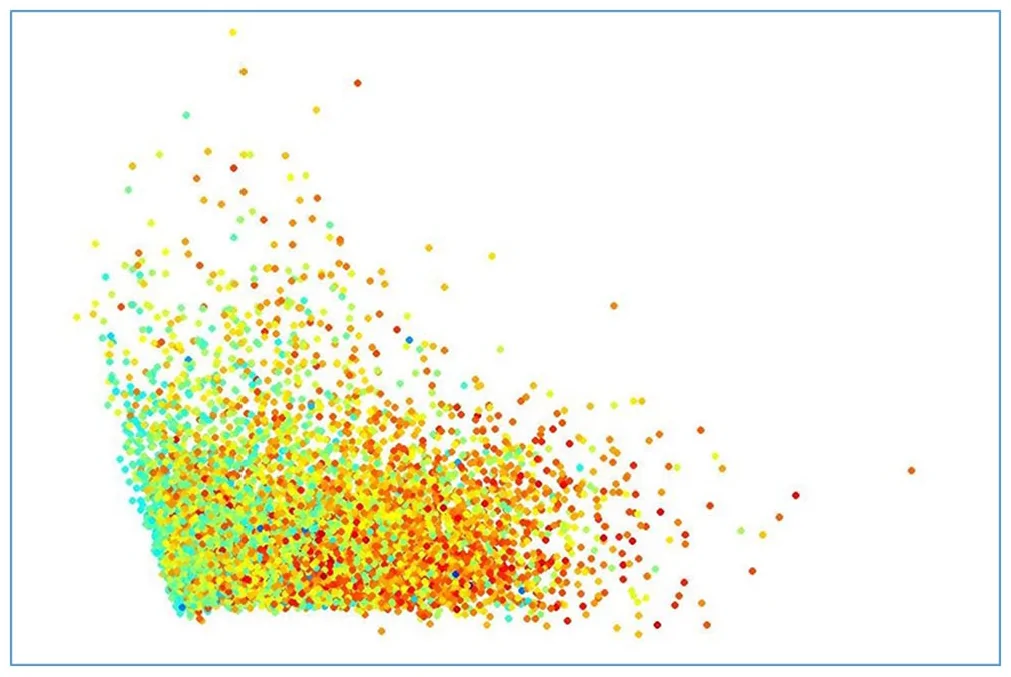
Color based on position in the diamond structure from pre-custody attributes, overlaid on the triangle structure of offenders (red—close to left-hand end of diamond, cyan—close to right-hand end of diamond).

The right-hand end of the triangle structure contains points corresponding to offenders who cause or become involved in various kinds of difficulties, challenges or concerns while in custody. We now explore whether it is possible to predict such offenders based on values of their pre-custody attributes. This would enable Corrections personnel to make more-refined assessments of risk on intake. Figure 6 shows the triangle structure associated with in-custody attributes overlaid with colors that represent how close each offender is placed toward the left-hand end of the diamond structure based on their pre-custody attributes. In other words, the color coding in the triangle is based on how far to the left each offender is placed in the diamond structure of figures such as Figure 2. The red points (offenders who have affinity for violence, affinity for substance abuse, or both) cluster moderately toward the region of the triangle that is associated with difficulties, challenges and concerns while in custody.

Some of the pre-custody attributes whose inherent variations are associated with left-to-right variation in the triangle structure (and so predictive of difficulties, challenges and concerns in custody) are:

- negative attitudes to criminal justice system
- supportive of instrumental violence
- attitudes domain score
- disrespectful of property (weak)
- employment domain score (weak)
- aggression issues
- has low frustration tolerance (weak)
- difficulty setting goals
- displays narrow and rigid thinking
- community domain score
- personal domain score

This list contains attributes that are related to poor interpersonal skills and poor coping skills, and to antisocial attitudes and behavior, but not general lifestyle instability. In other words (and unsurprisingly) the propensity for difficulties, challenges and concerns while incarcerated is more closely related to having antisocial attitudes and behavior combined with poor interpersonal and coping skills; it is not related to general lifestyle instability factors.

Several predictive models (support vector machines, random forests, neural networks) were constructed using pre-custody attributes as inputs and predicting left-to-right position in the triangle (i.e., propensity for difficulties). These typically achieved prediction accuracies around 70% (depending on how the target property is coded) which is non-trivial. This suggests that assessments of future propensity for difficulties may be possible in practice.

Attribute selection associated with such predictors of issues in custody suggests that the best predictive attributes are (in order): Number of federal sentences, sentence number, number of index offenses, attitudes domain score, marital/family domain score, community domain score, associates domain score, employment domain score, substance abuse domain score, and personal/emotional domain score.

High values of the attributes number of federal sentence and sentence number are negatively predictive of issues in custody (i.e., the greater the number of sentences, the less likely difficulties, challenges and concerns are), while high values of the other attributes are positively predictive. The fact that the domain score attributes are so predictive shows that they are extremely good surrogates for the meta-properties that they are designed to represent.

#### Limitations

The data used in this study was pulled from CSC databases and it is possible that they are incomplete or inaccurate because of errors or misconceptions in the design of the database query. The criteria for some attributes may have changed over the period used. For example, the criteria for STG membership changed slightly in 2003; although attempts were made by CSC to compensate, we cannot gauge if this was completely successful.

Detection of STG membership may be incomplete, although Pyrooz et al. (2020) found that administrative datasets have good concordance with other measures of gang membership, for example self-reporting.

There may be biases introduced because STG offenders receive higher levels of supervision (Pyrooz & Mitchell, 2020) and so may seem to be involved in higher numbers of incidents. Although CSC staff are trained in the use of the criteria for each of the attributes collected, there are more than 500 and it seems inevitable that, at least at the margins, there may be inconsistencies in the values entered into the offender databases.

Clustering techniques such as singular value decomposition produce a representation of variation that is a low-dimensional representation of an inherently high-dimensional space spanned by the attributes. Although it guarantees the most faithful representation possible in low dimension, there are necessarily losses. Intepreting the clustering that describes the variation among offenders also requires a qualitative analysis, so that only terms such as “strong” and “weak” relationships among attributes can be concluded.

## Conclusions

The research literature broadly suggests that STG offenders will differ from the general offender population in the following ways:

- Younger—supported (see the age demographic distribution in Table 1);
- To have been arrested more often, but convicted less—not supported (no consistent differences in almost all crime and sentencing attributes)
- More concerned with status and dominance—no evidence since personal attributes are primarily about the individual rather than about his/her relationship to others, except for familial relationships;
- Dislike for authority—supported (differences in the attributes: negative attitudes to the criminal justice system, displays non-conforming attitudes toward society);
- Less conscientious—perhaps (small differences in attributes such as: time management skills are problematic, financial difficulties);
- More impulsive and manipulative—supported (differences in the attributes: impulsive, manipulates others to achieve goals);
- More violent—supported (differences in the attributes: supportive of instrumental violence, any violent offense);
- Less educated—no evidence;
- Employed less and, if employed, paid less—not supported (no consistent differences in attributes such as: unemployed at time of arrest, employment: unstable or absent work history)
- Issues with alcohol—not supported (no consistent differences in substance abuse alcohol attributes)

However, even when differences are supported by the data, most are small. It is also the case that STG offenders are less likely to have committed a sexual offense compared to the general offender population.

Nafekh and Stys (2004) suggested further work to examine whether gangs (which they defined as “members or associates of a criminal organization”) have characteristically different risk assessments. The findings in this article suggest that they do, but the effect is weak—for example, members of traditional organized crime groups do not have high affinities for violence or substance abuse, and there is no systematic variation in these properties for other security threat groups. The current work does not indicate that gang members are more likely to have issues in custody, which Nafekh and Stys suggested might be the case. They also suggested that the relationship of power and position within a gang to aspects of custody be explored, which we have done to an extent limited by the available data; but the relationship seems to be weak. Properties of individual offenders are the best predictors of difficulties, challenges and concerns while incarcerated, rather than whether or not they belong to a STG.

For the pre-custody attributes, STG offenders are less extreme in the diamond structure than the general offender population. This may perhaps be because individuals who occupy the extremes, either with high affinities or low affinities, find it difficult to interact with others, and, therefore, do not join, or are not accepted by, groups of any kind. For example, someone with an extremely high affinity for violence may be considered too dangerous to associate with. The dangerous offender values are lower for STG offenders than the general population, which provides some evidence to support this. Attributes that tend to have higher values for the STG offenders and are associated with affinity for violence are almost all personality related, suggesting that characteristics of the individuals themselves, rather than their social environment, drive their organizational membership.

The attributes that tend to have higher values for STG offenders and are associated with affinity for substance abuse are more behavioral, and it is less clear whether they are causal or consequential. The attributes that have higher values for the STG offenders and are associated with *both* affinities suggest that family properties are correlated with STG participation, but wider social properties are not. The collective domain attributes community function domain score, marital/family domain score, and personal/emotional domain score clearly capture important aggregate properties of offenders.

For the in-custody attributes, the differences between the two groups are small, both for the independent and dependent attributes. There appears to be no indication that STG offenders are involved in more difficulties, challenges and concerns than the general offender population. For STG offenders, there is little relationship between role as a group member and other attributes collected at intake. This is perhaps surprising, since it might have been expected that, for example, senior management of an organized crime group might be associated with particular constellations of attitudes to violence. The exceptions are traditional organized crime groups, where expectations of low affinity for both violence and substance abuse appear to be the norm. Difficulties, challenges and concerns in custody are associated with high affinity for violence, substance abuse or both. Since the collective attributes are strong predictors in this setting, it is likely that corrections staff are already using this insight informally, but there may be potential benefits to using it more explicitly.

Overall, a small number of attributes (negative attitudes to the criminal justice system, supportive of instrumental violence, all of the collective domain measures, aggression issues, has low frustration tolerance) dominate the visible differences between the two groups of offenders.

## Notes

1. In 2002, the definition of a criminal organization reduced the number of necessary participants from 5 to 3 (Section 467.1(1) of the Criminal Code of Canada, making it consistent with the UN Convention on Transnational Organized Crime, Article 2).
